# Vascular Complications Following Trans-Trochanteric Fracture: Case Report and Literature Review

**DOI:** 10.3390/reports8040191

**Published:** 2025-09-29

**Authors:** Robert Bot, Adrian Tirla, Simona Daniela Cavalu

**Affiliations:** 1 Doctoral School of Biomedical Sciences, Faculty of Medicine and Pharmacy, University of Oradea, P-ta 1 Decembrie 10, 410087 Oradea, Romania; robertbbot@gmail.com (R.B.); adriantirla18@gmail.com (A.T.); 2 Department of Preclinical Sciences, Faculty of Medicine and Pharmacy, University of Oradea, P-ta 1 Decembrie 10, 410087 Oradea, Romania

**Keywords:** proximal femur fracture, iatrogenic vascular injury, profunda femoris artery, trans-trochanteric fixation

## Abstract

**Background and Clinical Significance**: Vascular complications occurring in the context of trans-trochanteric fractures are rare (mean incidence 0.2–0.5%) but can be fatal if not recognized and treated promptly. Most of the previously reported vascular injuries are iatrogenic, and various mechanisms of injury and producing agents have been reported. **Case Presentation**: We present a rare but severe vascular complication following proximal femur fracture fixation in the case of a 77-year-old patient, specifically, a deep femoral artery injury after DHS osteosynthesis. CT angiography identified the lesion in the territory of the profunda femoris artery, precisely at the level of the most distal screw, suggesting over-drilling as the underlying cause. The case is presented in the context of a literature review, updating the most important features of the vascular complications, incidence, diagnosis and treatment. **Conclusions**: This case highlights the critical role of early diagnosis and prompt interdisciplinary collaboration between orthopedic and vascular surgeons in managing iatrogenic vascular complications, achieving a favorable outcome.

## 1. Introduction and Clinical Significance

The elderly are more prone to fractures of the proximal femur (trochanteric fractures), undergoing hip replacement as a standard surgical treatment [1,2]. Vascular complications and injuries occurring during the course of internal fixation, manipulation and surgical procedures are rare, as most reports indicate an incidence of 0.2–0.5% [1,2,3], pseudoaneurysm being the predominant lesion type (67.03%) [4]. Approximately 80% of the lesions described in the literature affect the deep femoral artery [1,3,5,6], justifying the special attention that has to be paid to this vessel. Demographic changes and the increasing number of orthopedic interventions suggest that these complications will be encountered more frequently in practice [1,7]. Vascular injuries can occur either at the time of fracture, or during surgery, or even later, after weeks or months.

The present case illustrates a rare but severe vascular complication following proximal femur fracture fixation. Notably, intraoperative observation revealed that positioning the patient on the orthopedic traction table alters the anatomical relationship between the femoral shaft and the deep femoral artery, increasing the risk of injury during distal drilling. This observation emphasizes the need for heightened awareness, careful instrumentation, and appropriate imaging to prevent iatrogenic damage.

## 2. Case Presentation

A 77-year-old female patient was transported by ambulance to the emergency department, complaining of pain and functional impairment in the left hip, 2 days after falling from the same level at home. The patient had a medical history of arterial hypertension, mixed dementia, bronchial asthma, and chronic hepatitis C. The radiological examination reveals a per-trochanteric comminuted fracture with medial displacement of the lesser trochanter and atherosclerosis of the deep femoral artery. The patient was admitted to the orthopedic department of Bihor County Hospital, Oradea, Romania, and scheduled for surgery.

**Figure 1:**
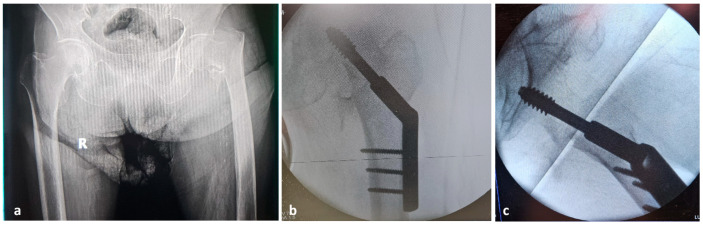
Radiologic images: (**a**) preoperative; (**b**) postoperative anteroposterior; (**c**) postoperative lateral view.

The patient was positioned in a dorsal recumbent position on the orthopedic surgery table, while axial traction and internal rotation of the left lower limb were practiced to reduce the fracture. Upon opening the fascia lata, a hematoma of ~400 mL was evacuated and significant active bleeding was observed. The involvement of a vascular surgeon was urgently required intraoperatively, but no significant vascular injury such as damage to the profunda femoris artery was observed. Only small-caliber vessels were identified as sources of active bleeding and were controlled by electrocautery and ligation, allowing hemostasis and enabling the fracture to be fixed with a dynamic hip screw (DHS) system, as presented in Figure 1.

**Figure 2:**
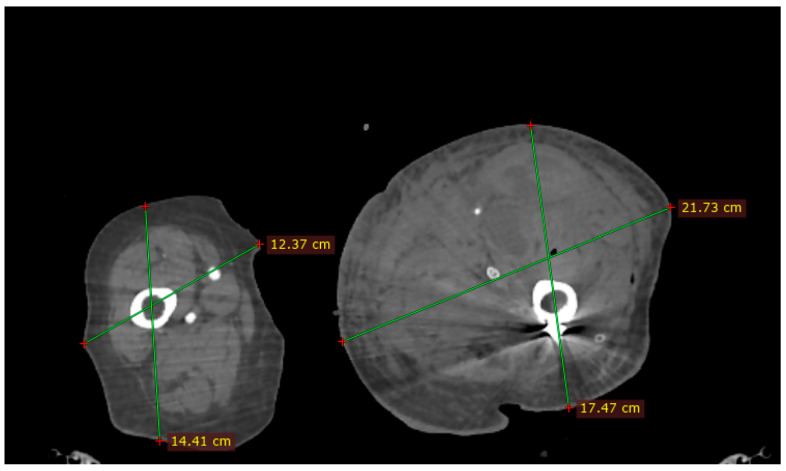
Angio CT image: Dimensional difference between left and right thigh.

A few hours after surgery, the patient’s general condition had deteriorated drastically, the left thigh increased significantly in volume and the hemoglobin level decreased from 14.6 g/dL (at the time of admission) to 5.7 g/dL. Angio-CT investigation was performed, identifying a lesion in the deep femoral artery, within the area of the most distal screw (Figure 2).

Emergency surgery re-intervention was performed. The profunda femoris artery was accessed and repaired through the same lateral incision used for fracture fixation. Bleeding was initially controlled by direct manual compression and electrocautery of small branches until the artery was clamped. No endovascular balloon occlusion was used. The lesion consisted of a perforation of the profunda femoris artery which was treated by primary repair (direct arteriorrhaphy) without ligation. Postoperative monitoring of arterial patency was performed clinically and with Doppler ultrasound during the entire hospitalization period. The patient was discharged in good condition. No information was available about the patient after discharge.

**Figure 3:**
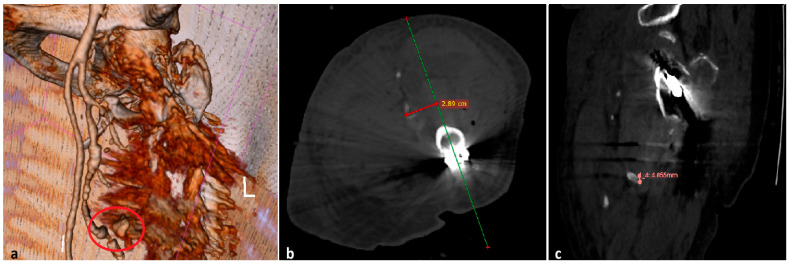
Angio-CT image investigation: (**a**) vascular injury associated with contrast medium extravasation (red circle); (**b**) the distance between the screw trajectory (green line) and the profunda femoris artery lesion, axial view (red line); (**c**) the distance between the vascular lesion and screw direction, sagittal view.

In order to identify the causes of the complication, the Angio-CT images were analyzed using 3D Slicer 5.0.3 and RadiAnt DICOM Viewer 2025.2 software (Figure 3). We identified a spatial separation between the screw direction (which corresponds to the drill trajectory) and the location of the vascular lesion. In the axial plane, a perpendicular line drawn from the screw trajectory to the site of the profunda femoris artery lesion measured 28.90 mm. In the sagittal plane, a vertical line drawn from the lesion to the screw trajectory showed a distance of 4.85 mm. These findings suggest that the geometry of the anatomical relationship between the femoral diaphysis and the profunda femoris artery may have been altered during fracture reduction maneuvers on the orthopedic traction table, bringing the artery into closer proximity to the femoral cortex than under normal conditions.

## 3. Discussion

The fixation of proximal femoral fractures and the management of their associated complications continue to be a subject of great interest to clinicians, presenting a high risk of morbidity and mortality. Often, these vascular complications involve the deep femoral artery, as a consequence of protrusion of various surgical instruments, retractor manipulation, bone fragment displacement, etc. Most of the reported vascular injuries are iatrogenic, while various mechanisms of injury and producing agents were reported. Table 1 summarizes the most relevant studies presented in the literature, highlighting the details of mechanisms and causes of lesions, types of lesions, anatomical location, implant types and treatment techniques.

In our case, the length of the distal screw was verified and found to be correct, thus excluding direct perforation of the profunda femoris artery by the screw itself. During the procedure, Farabeuf retractors were used and were consistently positioned lateral to the femoral diaphysis. Based on our measurements, the combination of the pelvic support compressing the medial aspect of the thigh, axial traction and internal rotation may have displaced the profunda femoris artery approximately 28.9 mm anteriorly and 4.85 mm proximally. Lateral displacement could not be determined. These measurements were derived from the distance between the vascular lesion and the trajectory of the drill bit at the level of the most distal screw.

Two main mechanisms of vascular injury after proximal femur fracture fixation have been described in the literature: displaced fragments of the lesser trochanter and iatrogenic trauma from surgical instruments, drill bits, or screws. In our case, overdrilling during DHS fixation was the mechanism leading to the profunda femoris artery injury. Nevertheless, displaced bone fragments can also injure adjacent vessels, and both mechanisms should be borne in mind during surgical planning and intraoperative vigilance.

In our literature review, we included patients reported in the literature who experienced vascular injuries associated with surgical fixation of proximal femur fractures. Specifically, we considered cases of intertrochanteric, subtrochanteric, femoral neck, and combined trochantero-diaphyseal fractures and subsequently developed an arterial complication. Both iatrogenic and fracture-related vascular injuries were included, proving that the fracture was located in the proximal femur.

We excluded all patients who did not experience a proximal femur fracture, as well as those with periprosthetic fractures, isolated traumatic pseudoaneurysms without fracture, and cases in which the fracture location or mechanism of injury could not be clearly determined. Duplicate cases that had already been reported earlier were also excluded to avoid double-counting.

As shown in Table 1, most vascular injuries associated with proximal femur fracture fixation were reported in intertrochanteric fractures treated with either dynamic hip screws or intramedullary nails [1,2,3,4,5,6,8,9,10,11,12,13,14,15,16]. The deep femoral artery was by far the most frequently affected vessel, accounting for more than half of all lesions, followed by the superficial and common femoral arteries [1,4,6]. Intrapelvic injuries were uncommon (<10%). The majority of cases were iatrogenic, mainly related to over-drilling or excessively long screws [12,13,14,16,17,18], while displaced lesser trochanter fragments were also an important cause [1,6,19,20,21]. Pseudoaneurysms represented the predominant type of vascular lesion, whereas arteriovenous fistulas were rare but well-documented, usually between the deep femoral artery and vein [4,8,12,13,15,16,18,20,21,22,23,24,25,26,27,28,29,30]. The International Surgery Journal (2023) also reported a profunda femoris artery pseudoaneurysm following dynamic hip screw fixation for an intertrochanteric fracture [31]. Additional recent case reports also confirm pseudoaneurysms as the most frequent vascular lesion after hip fracture fixation [12,14,29]. Overall, almost all patients required surgical or endovascular repair, with conservative management being exceptional [1,2,3,4,5,6,8,9,10,11].

### 3.1. Clinical and Paraclinical Diagnosis

Clinical alarm signs included cutaneous pallor, expanding hematoma with dimensional increase in the thigh, intense pain, local temperature change and, sometimes, the presence of a pulsatile mass [1,2,4,5,8]. Acute hemorrhage is manifested by hypotension and tachycardia, but patients treated with beta-blockers may have subtle signs [2,6]. Pseudoaneurysms may be silent and are often diagnosed late; they may cause progressive anemia, thigh edema, and chronic pain [1,3,4,9,15,18,28,29,30,32]. Investigation methods ranged from Doppler ultrasound and CT angiography to diagnostic and therapeutic angiography [1,9,20,33].

### 3.2. Treatment and Prognosis

Treatment strategies for vascular injuries associated with proximal femur fractures vary widely, ranging from conservative management (compression or observation) to complex surgical or endovascular interventions. The most frequently reported approaches included open surgical repair, vascular ligation, arterial suturing, endovascular coil embolization, and stent-graft placement. In several cases, thrombin injection guided by Doppler ultrasound has also been successfully employed [2,9,10,19]. Segal et al. [2] reported various endovascular techniques such as ultrasound-guided thrombin injection and coil embolization, including cases managed without endovascular intervention. Neubauer et al. [20] observed a complete recovery in 66% of patients, although the complication rate remained substantial. Across the reviewed literature, endovascular embolization emerged as the most frequently used therapeutic approach, particularly in the treatment of iatrogenic pseudoaneurysms [3,4,5,6,8,9,10,19,32,33,34,35,36,37]. Open surgical treatment was primarily indicated in cases of delayed diagnosis, ongoing bleeding, or failed endovascular management. Despite therapeutic advances, the overall morbidity and mortality associated with these vascular complications remain high, with a combined rate of approximately 18% [2,20].

### 3.3. Risk Factors

Anatomical and procedural factors have been associated with an increased risk of vascular injury following proximal femur fracture fixation. The deep femoral artery and its branches, due to their close anatomical proximity to the femoral shaft and lesser trochanter, are particularly vulnerable during drilling, screw placement, or nail insertion [1,2,37]. Risk is further elevated in cases involving displaced lesser trochanter fragments or fragment migration, which may cause direct arterial laceration [3,5,32,34]. Additional procedural risks include over-drilling, excessive screw length, incorrect trajectory, and implant mispositioning [1,10,33]. Several recent case reports illustrate these complications in contemporary practice, including delayed pseudoaneurysm of the deep femoral artery after intertrochanteric fracture fixation [38,39,40], pseudoaneurysm of the lateral femoral artery after pertrochanteric fracture fixation [41], and profunda femoris artery injury following proximal femoral fracture fixation [42]. Similar iatrogenic injuries have also been described, including a guide pin–related vascular complication and a profunda femoris artery pseudoaneurysm secondary to a displaced lesser trochanter fragment treated by coil embolization [41,43].Patient-related factors such as advanced age, severe osteoporosis, pre-existing vascular disease, and anticoagulant use may also contribute to vessel fragility and delayed diagnosis [1,2,4]. Inadequate intraoperative fluoroscopic control and lack of awareness of arterial landmarks further increase the likelihood of iatrogenic injury [11,19,37]. A comprehensive understanding of femoral vascular anatomy, meticulous surgical technique, and proper implant selection are essential to minimize these complications.

Among the 209 patients analyzed, 200 vascular injuries (95.69%) were considered iatrogenic. Of these, 28.71% (n = 60) were caused by bone fragments, most frequently the lesser trochanter (96.67% of cases). In 32.54% cases (n = 68), vascular damage resulted from uncontrolled drill bit advancement or the use of excessively long screws. Additional reported mechanisms included traction on the orthopedic table (4 cases), insertion of guidewires or reamers (8 cases) [22], the use of a retractor (1 case), ender elastic nails (1 case), an external fixator screw (1 case), and intrapelvic migration of screws (4 cases). In 53 patients, the precise cause could not be definitively identified, although uncontrolled drilling was the most frequently suspected mechanism [1,2,3,4,5,6,8,9,10,11]. A combined pseudoaneurysm and arteriovenous fistula of a branch of the profunda femoris artery after hip fracture fixation, successfully treated by endovascular embolization, was recently reported by Castelli Jr et al. (2022), illustrating the spectrum of vascular lesion types encountered [44].

### 3.4. Preventive Considerations

1. Preoperative vascular assessment—Patients with visible arterial calcifications on radiographs or known vascular comorbidities may benefit from preoperative vascular imaging, such as Doppler ultrasound or CT angiography, to evaluate arterial proximity and fragility [1,2,37].
2. Management of the lesser trochanter fragment—In cases with significant medial displacement, surgical reduction or excision of the lesser trochanter can reduce the risk of delayed arterial erosion, particularly of the deep femoral artery [3,5,32].
3. Instrumentation control—The use of drill stops, proper intraoperative measurement of screw length, and multiplanar fluoroscopic verification during implant placement are essential to prevent arterial penetration [1,10,33].
4. Postoperative surveillance—A sudden drop in hemoglobin levels, expanding hematoma, or persistent thigh pain should prompt immediate imaging evaluation (Doppler ultrasound or CT angiography). Clinicians should also be aware that beta-blocker therapy may mask tachycardia, potentially delaying recognition of internal bleeding [2,20].
5. Interdisciplinary collaboration—Early involvement of vascular surgeons and imaging specialists is strongly recommended in suspected or confirmed cases to ensure timely diagnosis and appropriate management [9,19,20].

### 3.5. Limitation

One limitation of our report is that we did not directly measure or demonstrate the displacement of vessels and soft tissues under traction table positioning. Future cadaveric or imaging-based modeling studies simulating standard positions on the traction table would be valuable to quantify such displacements and to better understand the risk of vascular injury during proximal femur osteosynthesis. These aspects would be valuable from didactic and educational perspective.

## 4. Conclusions

Vascular injuries associated with proximal femur fracture fixation are rare but potentially life-threatening events. The profunda femoris artery and its branches are particularly vulnerable due to their close anatomical relationship with the femoral cortex, surgical implants, and displaced fracture fragments—especially the lesser trochanter. Iatrogenic injury may occur intraoperatively through uncontrolled drilling or improper screw placement, or postoperatively due to secondary displacement or hardware migration.

This case presentation underscores the critical role of detailed preoperative planning, precise surgical technique, and high vigilance during and after surgery. Early recognition of vascular injury—guided by clinical signs such as unexplained anemia or expanding hematoma, and confirmed by Doppler ultrasound or CT angiography—is essential for timely intervention and favorable outcomes. Importantly, our findings suggest that intraoperative maneuvers, such as axial traction and internal rotation on the orthopedic traction table, may significantly alter the spatial relationship between the profunda femoris artery and the femoral diaphysis. This positional shift can bring the vessel closer to the drill or screw trajectory, increasing the risk of vascular injury. Surgeons should be aware of both anatomical and dynamic risk factors, particularly in elderly patients with fragile vasculature or atypical fracture patterns.

## Figures and Tables

**Table 1:** Summary of the most relevant studies reporting vascular injuries associated with trochanteric fractures.

First Author/Year/Reference	Article Type/Total Nr. of Cases/Incidence	Fracture Type (n)	Fracture Treatment (n)	Type of Lesion (n)	Location (n)	Mechanism of Injury (n)	Diagnosis (n)	Vascular Treatment (n)	Outcome (n)
Barquet et al., 2015 [1]	Systematic review/ 182/0.49%	Trochanteric (n = 132) Subtrochanteric (n = 12) Trochantero-diaphyseal (n = 5) Femoral neck (n = 28) Proximal femur- unspecified (n = 5)	Non-surgical (n = 3); Screws (n = 6); Pins (n = 2); Nail (n = 3); Nail plate (n = 19); Sliding nail plate (n = 1) SHS (n = 71); SHS + stabilizing plate (n = 1); SHS + antirotation screw (n = 1) Valgus osteotomy + SHS (n = 1) DLBP Gannet (n = 1); PCCP (n = 1) Ender nails (n = 5) Cephalomedullary nail -short (n = 45) Cephalomedullary nail -long (n = 9) Cephalomedullary nail long + cerclage (n = 1); Intramedullary nail- unspecified (n = 2); Gliding nail/revision (n = 1); Dynamic condylar screw/revision (n = 1); 958 angled blade plate (n = 2); Gamma nail-short (n = 2); PFN/PFNA (n ≥ 3) ORIF/undetermined (n = 3)	Pseudoaneurysm (n = 130); Laceration (n = 42); Thrombosis (n = 31); Arteriovenous fistula (n = 5); Intimal flap tear (n = 2); Compression (n = 1); Hematoma (n = 1)	Deep femoral artery (n = 129); Superficial femoral artery (n = 18); External iliac artery (n = 8); Internal iliac artery (n = 1); Common femoral artery (n = 5); Medial circumflex artery (n = 1); Lateral circumflex artery(n = 4); Obturator artery (n = 3); Popliteal artery (n = 4); Superior gluteal artery (n = 2); Common iliac vein (n = 1); External iliac vein (n = 3); Common femoral vein (n = 2); Unspecified vessel (n = 1)	Lesser trochanter fragment (n = 74); Overshot drill bit (n = 43); Protruding screw (n = 27); Traction/orthopedic table (n = 12); Retractor (n = 7) Other bone fragment (n = 9); Guide wire/reamer (n = 2); Implant migration (n = 5); Ender nails (n = 1); External fixator screw (n = 1); Cerclage wire(n = 1)	Angiography (n = 92); CT/CTA (n = 41); Ultrasound (n = 31) MRI (n = 2); Venography (n = 4); X-rays (n = 2) Intraoperative finding (n = 10)	Artery repair (n = 54); Artery ligation (n = 38); End-to-end anastomosis (n = 6); Vein repair/ligation (n = 7); Bypass graft (n = 3); Endovascular embolization (n = 38); Stent (n = 12); Thrombectomy/angioplasty (n = 4) Bone fragment removal (n = 5); Conservative (n = 9); Amputation (n = 2); Death before treatment (n = 4)	Uneventful recovery (n = 140); Death-vascular-related (n = 5); Death -unrelated (n = 3); Amputation (n = 2); Infection- controlled (n = 2); Infection-persistent (n = 1); Gangrene/toe resection (n = 2); Occlusion (n = 1); Vein thrombosis (n = 1); Neurological impairment (n = 2); Functional limitation (n = 1); Girdlestone procedure (n = 1); Other sequelae (n = 21)
Agustín et al., 2022 [7]	Systematic review/ 40/20 unique cases	Intertrochanteric (n = 15); Subtrochanteric (n = 3); Femoral neck (n = 1); Pathological intertrochanteric (n = 1)	DHS/SHS (n = 9); PFN/PFNA (n = 7); Gamma nail (n = 2); Arthroplasty (n = 1) Other (n = 1)	Pseudoaneurysm (n = 17); Laceration (n = 2); Thrombosis (n = 1)	Deep femoral artery (n = 15); Superficial femoral artery (n = 2); External iliac artery (n = 1); Obturator artery (n = 1); Lateral circumflex femoral artery (n = 1)	Protruding screw (n = 7); Overshot drill bit (n = 5); Lesser trochanter fragment (n = 3); Implant migration (n = 2); Guide wire (n = 1); Other (n = 2)	Angiography (n = 7); CT/CTA (n = 5); Ultrasound-based (n = 6); MRI (n = 1); Intraoperative finding (n = 1)	Surgical repair/ligation/graft (n = 12); Endovascular (n = 6); Conservative (n = 1); Amputation (n = 1)	Uneventful recovery (n = 16); Death (n = 1); Chronic pain/sequelae (n = 1); Recurrence/reintervention (n = 1) Not specified (n = 1)
Segal et al., 2017 [2]	Retrospective cohort study/ 1469/0.2% (3 cases)	Intertrochanteric (n = 3)	Gamma nail (n = 2); DHS (n = 1)	Active bleeding/pseudoaneurysm (n = 3)	Deep femoral artery (n = 3)	Overdrill (n = 2) Lesser trochanter fragment (n = 1)	CTA (n = 3)	Conservative (n = 1); Coil embolization (n = 1) Thrombin injection (n = 1)	Uneventful recovery (n = 3)
Kim et al., 2020 [8]	Case report and literature review/1/-	Intertrochanteric (n = 1)	Gamma nail (n = 1)	Arteriovenous fistula (n = 1)	Common femoral artery, superficial femoral artery, deep femoral artery and femoral vein (n = 1)	Overdrill/implant manipulation (n = 1)	Doppler ultrasound initial (n = 1); CTA confirmation (n = 1)	Open surgical repair with vein graft (n = 1)	Uneventful recovery (n = 1)
Orapiriyakul et al., 2022 [4]	Case report and literature review/1/-	Intertrochanteric (n = 1)	PFN + cerclage wire (n = 1)	Pseudoaneurysm (n = 1)	Branch of deep femoral artery (n = 1)	Cerclage wire (n = 1)	Doppler ultrasound -initial (n = 1); CTA confirmation (n = 1)	Open surgical repair with direct suture (n = 1)	Uneventful recovery (n = 1)
Samaan et al., 2023 [6]	Case report/1/-	Intertrochanteric (n = 1)	DHS (n = 1)	Laceration/perforation with active bleeding (n = 1)	Deep femoral artery (n = 1)	Long self-tapping DHS screws (n = 1)	Doppler ultrasound- false negative (n = 1) Intraoperative finding (n = 1)	Arteriorrhaphy (direct suture) + screw removal (n = 1)	Uneventful recovery (n = 1)

Legend: n = number of cases; SHS—Sliding hip screw; DHS—Dynamic hip screw; DLBP—dynamic locking blade plate; PCCP—Percutaneous compression plate; PFN—Proximal femoral nail; PFNA—proximal femoral nail antirotation; ORIF—Open reduction internal fixation; CT—Computed tomography; CTA—Computed tomography angiography; MRI—Magnetic Resonance Imaging.

## Data Availability

The original contributions presented in this study are included in the article. Further inquiries can be directed to the corresponding author.
